# Plant-Produced Chimeric Hepatitis E Virus-like Particles as Carriers for Antigen Presentation

**DOI:** 10.3390/v16071093

**Published:** 2024-07-08

**Authors:** Eugenia S. Mardanova, Egor A. Vasyagin, Kira G. Kotova, Gergana G. Zahmanova, Nikolai V. Ravin

**Affiliations:** 1 Institute of Bioengineering, Research Center of Biotechnology of the Russian Academy of Sciences, 119071 Moscow, Russia; mardanovaes@mail.ru (E.S.M.);; 2 Department of Molecular Biology, University of Plovdiv, 4000 Plovdiv, Bulgaria; 3 Department of Technology Transfer and IP Management, Center of Plant Systems Biology and Biotechnology, 4000 Plovdiv, Bulgaria

**Keywords:** virus-like particle, hepatitis E virus, transient expression, *Nicotiana benthamiana*, viral vector, vaccine, M2e peptide, influenza

## Abstract

A wide range of virus-like particles (VLPs) is extensively employed as carriers to display various antigens for vaccine development to fight against different infections. The plant-produced truncated variant of the hepatitis E virus (HEV) coat protein is capable of forming VLPs. In this study, we demonstrated that recombinant fusion proteins comprising truncated HEV coat protein with green fluorescent protein (GFP) or four tandem copies of the extracellular domain of matrix protein 2 (M2e) of influenza A virus inserted at the Tyr485 position could be efficiently expressed in *Nicotiana benthamiana* plants using self-replicating vector based on the potato virus X genome. The plant-produced fusion proteins in vivo formed VLPs displaying GFP and 4M2e. Therefore, HEV coat protein can be used as a VLP carrier platform for the presentation of relatively large antigens comprising dozens to hundreds of amino acids. Furthermore, plant-produced HEV particles could be useful research tools for the development of recombinant vaccines against influenza.

## 1. Introduction

Virus-like particles (VLPs) are actively used in preventive medicine. A wide range of VLP-based candidate vaccines has been developed for immunization against various infectious agents [1,2,3]. VLP represents a self-assembled structure, which consists of multiple monomeric units, that imitates the virus’ morphology [4]. Meanwhile, VLPs do not include viral nucleic acid, and in consequence, they are non-infectious and safe. VLPs can stimulate both humoral and cellular immune responses [5,6]. Accordingly, VLP vaccines combine both safety and ability to induce an immune response through their size, shape and surface [7]. Moreover, their stability provides ease in storage and transportation.

Several VLP-based vaccines are currently commercially available with other vaccines in various phases of clinical trials [1]. VLPs can act as vaccines by themselves and furthermore they can be used as carriers for other protein antigens. The clustering of an antigen on the surface of VLP greatly enhances induction of an immune response [8]. Thus, VLP-based carriers can provide the possibility to increase the immunogenicity of the foreign antigens linked to them [8,9]. However, the attachment of a foreign peptide to the VLP-forming protein may interfere with self-assembly [10]. Properties of the antigen, such as its length, particular amino acid sequence, hydrophilicity and hydrophobicity, can affect the VLPs assembly. The antigen insertion point is essential for correct assembly of VLP and presentation on the surface.

VLPs can be produced in various expression systems including bacterial cells [11,12,13], yeasts [14], insect cells [15], plants [9,16,17], and mammalian cells [18,19]. Plants are actively used as a biomanufacturing platform for recombinant protein production [20,21]. The advantages of this expression system include easy scale-up, cost effectiveness, and the absence of common pathogens in animals and plants. In addition, plants are capable of post-translational modifications, although there are significant differences in this regard between plants and mammalian cells [22,23,24]. The plant expression system is already being used to produce vaccines on an industrial scale. For example, the COVIFENZ^®^ COVID-19 vaccine produced in *Nicotiana benthamiana* has been approved in Canada, and several plant-made influenza vaccines have undergone clinical trials [25].

In this study, we tested the potential of hepatitis E capsid protein as a carrier of foreign antigens in vaccine development. Truncated hepatitis E capsid protein comprising 110–610 a.a. has been efficiently expressed in *N. benthamiana* plants and formed VLPs with diameters varying from 19 to 31 nm [24,26]. The results from different studies revealed that the truncated hepatitis E virus (HEV) capsid protein expressed in heterologous expression systems can be self-assembled into VLPs with T1 symmetry, indicating that some of the recombinant HEV particles deviated in size [27,28]. The computational modeling of the full-length HEV coat protein (genotype 3) revealed three regions, which are most suitable for peptide insertion (a.a. 480–490, 550–565, and 580–590) and correspond to three loops on the surface spike [29]. The 15 a.a. long antigenic p18 peptide from the V3 loop of HIV-1 gp120 was inserted after residue Tyr485 in the P-domain of HEV CP [30]. The fusion protein was able to form VLPs, which did not react with anti-HEV antibodies. Thus, the residue Tyr485 is a promising candidate for insertion of at least short peptides without interfering with capsid assembly [30]. Later, recombinant HEV capsid (110–610 a.a.) with M2e peptide of influenza A virus (23 a.a.) inserted at the Tyr485 position was expressed in plants at high yield and was found to form nanosized VLPs in vivo [31]. However, the HEV capsid with the insertion of a longer peptide, the receptor binding domain (RBD) of the SARS-CoV-2 spike glycoprotein (206 a.a.) was expressed at a very low level and was found to be mostly insoluble [31].

We analyzed the possibility of using the HEV capsid protein as a carrier of longer polypeptides and whether chimeric VLP can be obtained in plants. As the first model protein for insertion, we chose the green fluorescent protein (GFP). The GFP used in the study was 239 a.a. long and had a mass of 27 kDa. It is often used as a model protein in VLP design and has been efficiently expressed in plants up to several mg per gram of plant biomass [32,33,34,35,36].

As the second model protein for insertion, we chose the ectodomain of the M2 protein (M2e) of influenza A virus, consisting of 23 a.a. This peptide is highly conserved and may serve as a basis for the creation of “universal” vaccines against a broad spectrum of influenza strains. However, the small size of M2e leads to low immunogenicity and, consequently, weak protective properties [37]. This problem can be solved by linking M2e to different carriers, particularly VLPs [38,39,40,41,42]. The density of M2e presentation on the surface of the VLP is important for the efficacy of the vaccine candidate. Experimental influenza vaccines based on VLPs containing the M2e peptide in the immunodominant loop region of the hepatitis B core (HBc) antigen revealed that the protective capabilities of the M2e-HBc particles correlate with the copy number of M2e. Particles formed by the HBc peptide linked to four copies of M2e were more immunogenic than particles formed by HBc carrying one or two copies [43]. Therefore, in this study, four copies of M2e peptide were used to enhance the immune response.

The selected M2e peptide was derived from the swine influenza A virus, which causes a highly contagious infection, with 10–15% lethality among unvaccinated animals [44]. The influenza A infection progresses rapidly and can lead to feverish abortions in sows, infertility, complications from secondary infections, and the development of chronic diseases [45]. As a result, infection with swine influenza A virus can cause significant economic losses due to slowed weight gain and increased days-to-market weight attainment. Thus, the development of a vaccine against swine influenza A with a broad strain range is an important task.

Here, we designed fusion proteins comprising GFP or four copies of M2e peptide inserted into HEV capsid protein. The proteins were transiently produced in plants using a viral-based expression vector. We have shown that GFP insertion, as well as insertion of four copies of M2e, does not interfere with VLP assembly. Thus, the truncated HEV capsid protein (110–610 a.a.) can be used as a carrier of long antigens for the development of vaccines.

## 2. Results

### 2.1. Viral Vectors for the Expression of HEV/GFP and HEV/4M2e Hybrid Proteins in Nicotiana benthamiana

**Figure 1:**
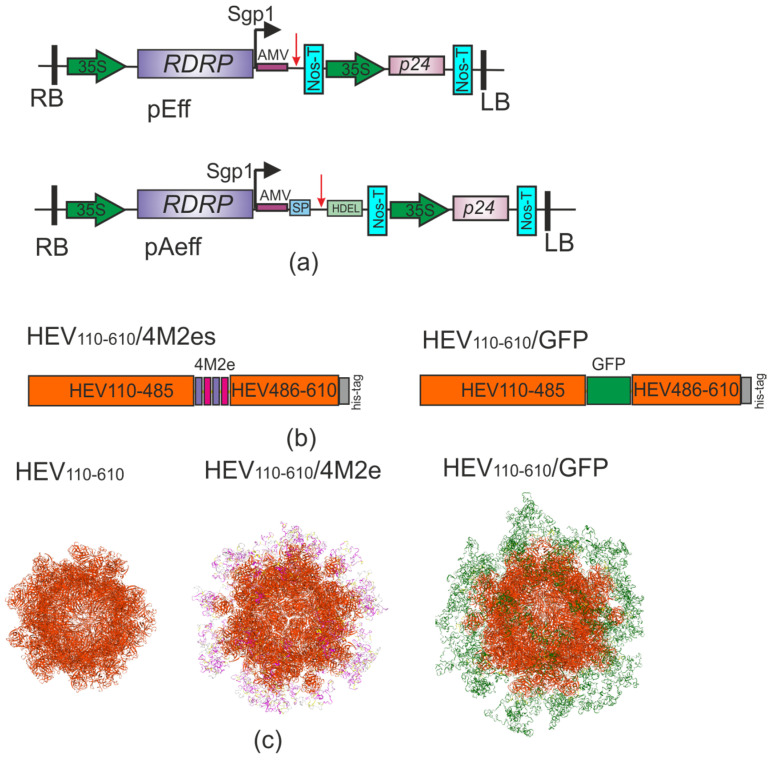
(**a**) Scheme of the expression vectors. RDRP, RNA-dependent RNA polymerase gene; Sgp1, the first promoter of subgenomic RNA of PVX; AMV, translational enhancer from alfalfa mosaic virus; 35S, promoter of the cauliflower mosaic virus RNA; Nos-T, terminator of the *A. tumefaciens* nopaline synthase gene; P24, suppressor of silencing from grapevine leafroll-associated virus-2; SP, signal peptide; HDEL, ER retention signal; RB and LB, the right and left borders of T-DNA region, respectively. The insertion position of the target gene I is indicated by the red arrow. (**b**) Scheme of the recombinant proteins. 4M2e, the sequence of four tandem copies of M2e peptide; GFP, green fluorescent protein; HEV, hepatitis E virus ORF2. (**c**) 3D modeling of virus-like particles formed by HEV ORF2 capsid, HEV/4M2e, and HEV/GFP proteins by SWISS MODEL.

Foreign peptides (four copies of M2e or GFP) were inserted into the HEV ORF2 capsid (110–610 a.a.) at the Tyr485 being flanked by GGGSG linkers to ensure proper protein folding (Figure 1). Tandem copies of M2e were also separated from each other by the same linkers. For the HEV/4M2e fusion we used four copies of the M2e peptide: two peptides corresponded to the consensus sequence of H1N1 and H1N2 of the swine influenza A virus, and two peptides corresponded to the consensus sequence of H3N2 of the swine influenza A virus. Their sequences differ from each other at one position.

SWISS-MODEL server [46] was used to predict the spatial structure of the hybrid proteins. The 3D structures of HEV/GFP and HEV/4M2e were predicted to assemble into 60 monomers VLPs, whereas GFP (green) and 4M2es (purple) were predicted to be exposed on the surface of the produced VLPs (HEV, orange) (Figure 1).

The pEff viral vector [36] was used to express the HEV, HEV/GFP and HEV/4M2e proteins in *N. benthamiana* plants. For the expression of HEV and HEV/4M2e, we additionally used the pAeff vector [31], enabling targeting of the expressed protein to the endoplasmic reticulum (ER). For this purpose, we added ER-targeting signal peptide at N-terminus and ER retention signal HDEL at the C-terminus of the fusion protein.

### 2.2. Expression of HEV/GFP and HEV/4M2e in Plants and Protein Purification

**Figure 2:**
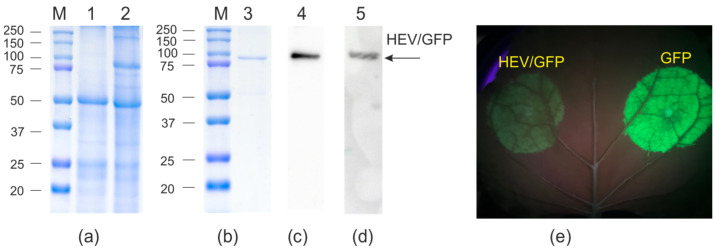
Expression of HEV/GFP protein in *N. benthamiana* plants. Coomassie brilliant blue-stained gel (**a**,**b**) and Western blotting with antibodies against GFP (**c**) and hexahistidine tag (**d**) of proteins isolated from plants. M, molecular weight marker (kDa); Lanes: 1, total proteins isolated from non-infiltrated leaf; 2, total proteins isolated from leaf infiltrated with pEff-HEV/GFP; 3–5, purified HEV/GFP protein. Position of HEV/GFP protein is shown by an arrow. Visualization GFP fluorescence in *N. benthamiana* leaves infiltrated with pEff_HEV/GFP and pEff-GFP in UV (**e**).

Recombinant binary vectors were introduced into the *A. tumefaciens* strain GV3101 by electroporation. *N. benthamiana* leaves were infiltrated with agrobacteria carrying the recombinant vectors. As a control for GFP fluorescence we used the pEff-GFP vector, enabling expression of GFP without a fusion partner [36]. GFP fluorescence in leaves was monitored by UV irradiation and quantitative fluorimetry. The pEff_HEV/GFP vector produced three–five-fold lower GFP fluorescence compared to that of pEff-GFP (Figure 2e).

The protein samples from the infiltrated zones were analyzed using SDS-PAGE and Western blotting. An SDS-PAGE analysis showed that HEV/GFP was efficiently synthesized, accounting for about 10% of the total plant protein (Figure 2a) and was partially observed in the soluble fraction (Figure S1). The maximal expression level of HEV/GFP protein was observed on the fourth day after infiltration (dpi) (Figure S2).

**Figure 3:**
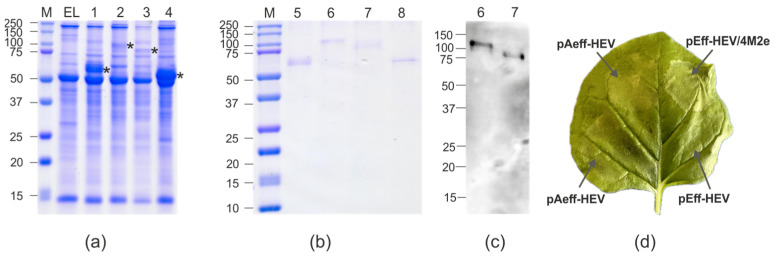
Expression of HEV and HEV/4M2e proteins in *N. benthamiana* plants. Coomassie brilliant blue-stained gel (**a**,**b**) and Western blotting with antibodies against M2e (**c**) of proteins isolated from plants. M, molecular weight marker (kD); EL, total proteins isolated from the non-infiltrated leaf; Lanes: 1, total proteins isolated from leaf infiltrated with pAeff-HEV; 2, total proteins isolated from leaf infiltrated with pAeff-HEV/4M2e; 3, total proteins isolated from leaf infiltrated with pEff-HEV/4M2e; 4, total proteins isolated from leaf infiltrated with pEff-HEV; 5, purified HEV protein (expression using pAeff vector); 6, purified HEV/4M2e protein (expression using pAeff vector); 7, purified HEV/4M2e protein (expression using pEff vector); 8, purified HEV protein (expression using pEff vector). Visualization of proteins synthesized in *N. benthamiana* leaves infiltrated with pAeff-HEV, pAeff-HEV/4M2e, pEff-HEV/4M2e and pEff-HEV (**d**). The positions of HEV and HEV/4M2e proteins in panel (**a**) are marked with an asterisk (*).

HEV/4M2e was expressed at the level of about 6–8% of total soluble plant protein (about 150–200 μg per 1 g of green leaf biomass) (Figure 3) and appeared to be mostly soluble (Figure S3). The control protein, HEV (110–610 a.a.), was expressed at the level of about 20% of the total soluble protein. The expression levels of these proteins did not differ between the vectors pEff and pAeff. We observed moderate necrosis in the zones infiltrated with agrobacteria carrying vectors encoding the HEV/4M2es protein that can be associated with toxicity of the corresponding protein (Figure 3c). The maximal expression level was observed on the third or fourth dpi.

The biomass of agroinfiltrated leaves was collected on the third–fourth dpi. Protein purification was carried out using metal-affinity chromatography under native conditions. Purified protein samples were characterized using SDS-PAGE and Western blotting (Figure 2 and Figure 3). The final yield of the hybrid proteins after purification was about 100 μg/g of green leaf biomass for HEV/GFP and 60–80 μg/g for HEV/4M2e, while the yield of HEV was about 200 μg/g of green leaf biomass. HEV/GFP protein was specifically revealed in Western blotting with the antibodies against GFP and the hexahistidine tag.

### 2.3. Plant-Produced HEV/GFP and HEV/4M2e Proteins Formed VLPs

**Figure 4:**
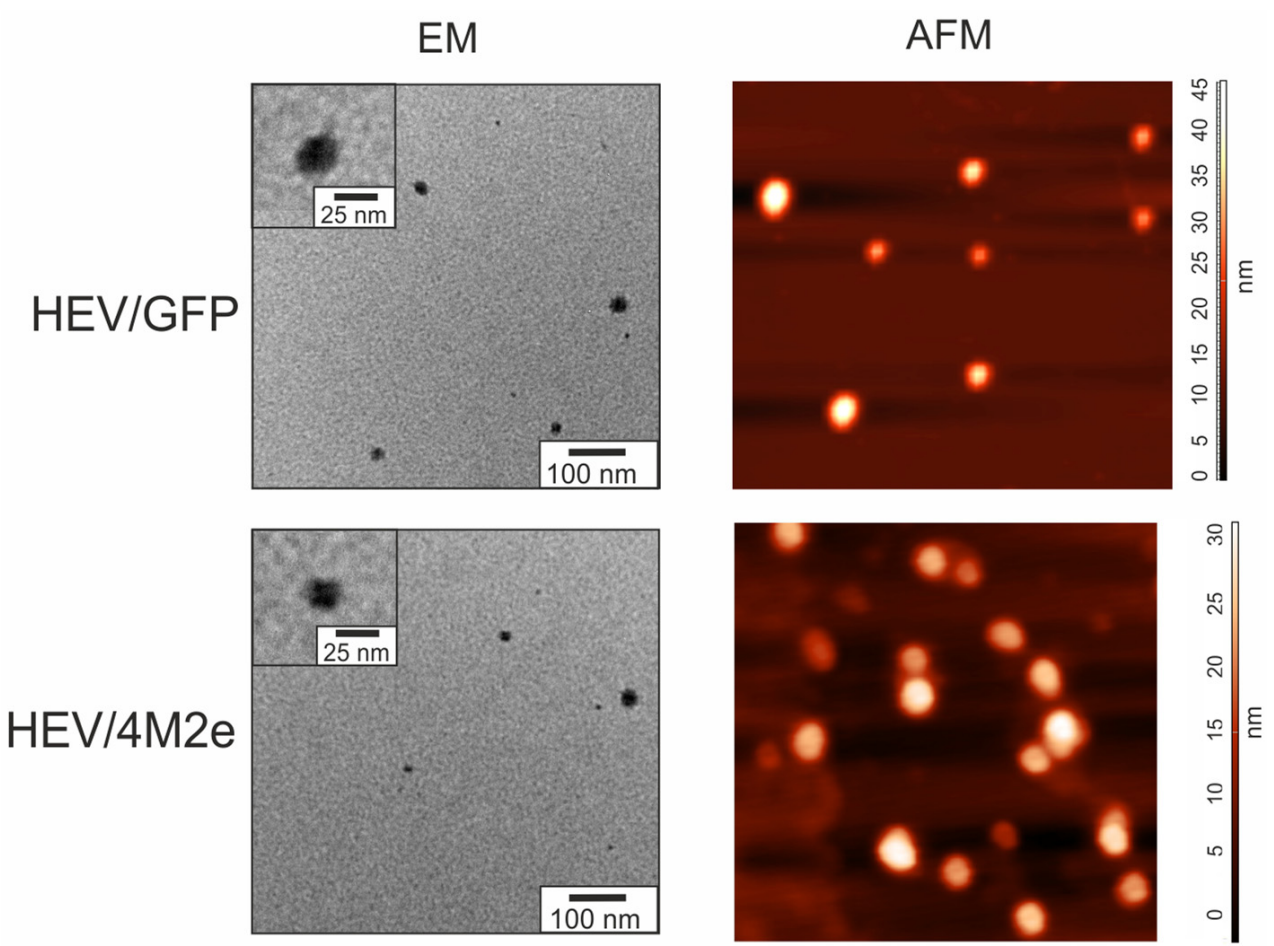
Analysis of virus-like particles formed by HEV/GFP and HEV/4M2e proteins by transmission electron microscopy (EM) and atomic force microscopy (AFM). The color scale shows particle height.

The assembly of the HEV/GFP and HEV/4M2e proteins into nanosized structures was analyzed by electron and atomic force microscopy. We observed heterogeneous particles by both methods (Figure 4).

According to transmission electron microscopy, both HEV/GFP and HEV/4M2e formed spherical particles with a diameter of about 21–24 nm (Table 1). The particle height, estimated by atomic force microscopy, was nearly the same for HEV/4M2e and slightly larger for HEV/GFP (Table 1, Figure S4). The data on the polydispersity index (PDI) indicated that HEV/GFP particles are more heterogeneous in size than that formed by HEV/4M2e.

### 2.4. Inserted Peptides Are Displayed on the Surface of VLPs

To ensure high efficacy of a VLP-based vaccine, it is important that the antigen is presented on the surface of the chimeric particle and not hidden inside. To analyze the position of the antigen on VLP, we tested its accessibility to specific antibodies using ELISA.

**Figure 5:**
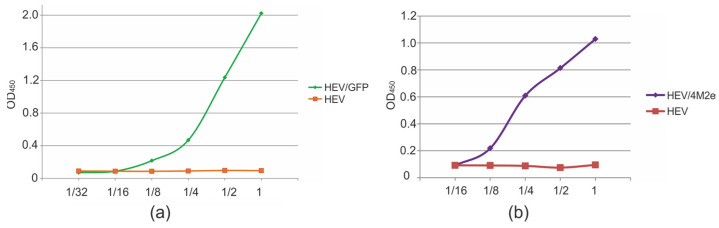
Antigenicity of the recombinant proteins. Two-fold dilutions of recombinant HEV, HEV/4M2e, and HEV/GFP proteins (starting from 10 μg/mL) were used to coat ELISA plates which were then probed with antibodies specific for GFP (**a**) or M2e (**b**).

ELISA plates, coated with serial dilutions of HEV/GFP, HEV/4M2es, and a control HEV protein, were probed with GFP- or M2e-specific monoclonal antibodies. HEV/GFP and HEV/4M2es reacted with anti-GFP and anti-M2e antibodies, respectively (Figure 5). These results confirmed the accessibility of the HEV/GFP and HEV/4M2es particles to antibodies against these antigens and suggest that the inserted proteins are exposed on the surface of the recombinant VLPs.

## 3. Discussion

Virus-like particles have gained widespread popularity as carriers of foreign epitopes in the development of vaccines against various diseases due to their convenience and effectiveness [1,9,11,14,18,47]. Various expression systems can be used for the production of recombinant VLPs [9,36,48]. Plant expression systems combine the simplicity and low cost of prokaryotic systems and the ability for post-translational modifications characteristic for eukaryotes, especially glycosylation, which cannot be realized by the prokaryotic cell [49]. In this study, we performed transient expression in plants using self-replicating viral vector based on the PVX genome.

It has been shown previously that a truncated version of the HEV coat protein (110–610 a.a.) can be expressed at high levels in plants and can form virus-like particles [24,50]. In addition, this variant of the HEV capsid can be used as antigen carrier in the plant expression system [24,31]. In terms of expression levels and presentation of foreign peptides in plants, HEV significantly exceeds another popular carrier for the presentation of foreign peptides, the HBc antigen [51]. In our previous studies, HBc and HBc-M2e fusions were expressed in *N. benthamiana* plants up to 5–10% of total protein [36], which is several times lower compared to HEV [24,50]. However, the capacity of the HBc antigen for foreign insertions is rather high, as demonstrated by successful insertions of the entire 238-aa GFP [52] and OspC protein of *Borrelia burgdorferi* [53]. In addition, previously we were able to obtain recombinant HBc particles carrying four copies of the influenza M2e peptide [43] from a bacterial expression system, while the expression of similar proteins in plants failed.

The HEV capsid could become a convenient carrier of foreign peptides that can be expressed at high levels in plants. However, such a carrier must tolerate insertion of large peptides (tens to hundreds of amino acids) without affecting the expression level and the ability of the fusion protein to self-assemble into VLPs. Therefore, in this study, we inserted longer peptides into the HEV capsid protein to investigate their effect on proper folding of the fusion protein and VLP formation. We inserted foreign polypeptides, GFP and four copies of the M2e peptide of the influenza A virus at position Tyr485 of the HEV capsid protein. In order to facilitate proper folding of the fusion proteins, GFP and four copies of the M2e peptides were separated from the HEV coat protein backbone by flexible glycine-rich linkers. The introduction of such linkers is especially important because when inserting an epitope at an internal position, rather than at the N or C terminus of the capsid protein, the linkers are required to ensure spatial proximity of the ends of the inserted peptide.

In this study, we showed that both the HEV/GFP and HEV/4M2es proteins can be efficiently expressed in plants and form VLPs in vivo; moreover, we observed GFP fluorescence, indicating the stability of the structural GFP organization. We showed that the hybrid proteins HEV/GFP and HEV/4M2e were able to bind Abs against GFP and M2e, respectively, indicating epitope display on the particle’s surface.

According to transmission electron microscopy, both HEV/GFP and HEV/4M2e formed heterogeneous particles with a diameter of 24 ± 8 nm and 21 ± 6 nm, respectively. Considering the PDI values, the particles could be considered as narrow to moderately disperse (PDI < 0.4) and HEV/GFP particles appeared to be more heterogeneous than particles made by HEV/4M2e. Likewise, “empty” HEV capsid protein (110–610 a.a.) produced in plants assembled into spherical VLPs varying from 19 to 31 nm in size [24]. Therefore, the heterogeneity of VLPs is not the result of the insertion of foreign proteins, but is a property of truncated HEV capsid itself. A comparable phenomenon was noted when pea enation mosaic virus 1 (PEMV1) capsid protein was expressed in plants [54].

The ratio of heights to diameters of VLPs, determined by AFMa, was around 0.12 for HEV/GFP and 0.21 for HEV/4M2e (Figure S5), suggesting that the particles are not solid bodies and likely contain internal empty cavities. HEV/GFP particles appeared to be “softer” consistently with their higher heterogeneity. However, these data should be considered with care since AFM technique offers an ability to measure, with a high degree of accuracy, only the heights of objects, while the lateral sizes appear to be enlarged due to a tip-related effect [55].

In our previous studies, we used HEV capsid protein (110–610 a.a.) for displaying a single copy of the M2e peptide [24,50]. The recombinant protein was efficiently expressed in plants and formed VLPs. However, immunization of mice with such particles induced only low levels of M2e-specific antibodies. The linking of several copies of M2e to a carrier protein was shown to increase the immune response to M2e and the protective capabilities of the candidate vaccine [43,56,57,58]. Another advantage of including multiple copies of the M2e peptides in VLPs is the ability to expand the strain specificity of the vaccine. Although the M2e peptide is highly conserved in human strains of influenza A virus, in strains of animal origin the M2e sequence may differ in several amino acids, and these differences affect the specificity of the immune response [59,60].

HEV/M2e protein comprising a single copy of M2e could be synthesized in plants accounting for about 10% of the total soluble protein [31] while the expression levels of HEV alone, without foreign peptides, is in the range of 10–20% [24,50]. Larger insertions moderately decreased the yield of recombinant HEV-based fusions: HEV/GFP was expressed at about 10% and HEV/4M2e at about 6–8% of total soluble protein. We anticipated that targeting the endoplasmic reticulum might increase the expression level or improve the solubility of the recombinant protein, but this was not observed. We have observed moderate necrosis in the zones infiltrated with agrobacteria carrying vectors encoding the HEV/4M2es protein. An increase in M2e copy number can lead to a toxic effect caused by the expression of a recombinant protein. It is likely that the toxicity of the HEV/4M2e protein also limits the level of expression achieved. Nevertheless, the final yield of HEV/4M2e protein after purification was about 60–80 μg per 1 g of leaf biomass. This yield is sufficiently high for protein production by a plant expression system.

## 4. Materials and Methods

### 4.1. Vector Construction

We used the self-replicating viral vector pEff [36]. Vector pEff_HEV/M2e encoding HEV 110–610 capsid with M2e peptide inserted at Tyr485 position [31] was used for cloning of the gfp gene. Gfp was obtained by PCR with primers GFP_GS_XhoI_F (ATCTCGAGGG AGGTGGATCT GGAGTGAGCA AGGGCGAGGA GCT) and GFP_GS_SnaBI_R (ATTACGTATC CAGAACCACC TCCCTTGTAC AGCTCGTCCA TGC) using pEff-GFP vector as a template. The PCR fragment was cloned into pEff_HEV/M2e instead of M2e resulting in the pEff_HEV/GFP expression vector.

For HEV/4M2e, we used four tandem copies of M2e peptide; two copies corresponded to the consensus sequence of H1N1 and H1N2 strains of swine influenza A virus (SLLTEVETPTRSEWECRCSDSSD), and two copies corresponded to the consensus sequence of H3N2 strains of swine influenza A virus (SLLTEVETPTRNEWECRCSDSSD). These consensus sequences were derived from multiple alignments of M2e of swine influenza A strains of the H1N1, H1N2, and H3N2 subtypes obtained using the MAFFT v.7.490 tool [61]. In order to facilitate folding of the hybrid protein, copies of M2e peptides were separated from each other by flexible glycine-rich linkers. The sequence encoding 4M2e was synthesized in vitro (Evrogen, Moscow, Russia) and cloned instead of the *gfp* gene in the pEff vector. The obtained vector was designated pEff-HEV/4M2es.

For HEV/4M2es expression, we additionally used pEff-based vector targeting the recombinant protein to the endoplasmic reticulum. For this purpose, the pEff vector was modified to encode the signal peptide (MIMASSKLLSLALFLALLSHANS) from *Phaseolus vulgaris* at the N-terminus and the endoplasmic reticulum retention signal sequence HDEL at the C-terminus of the target protein as described in [31]. The control HEV protein (110–610 a.a.) was expressed using expression vectors pEff and pAeff.

### 4.2. Agroinfiltration of N. benthamiana Plants

Plants were grown in a greenhouse under a 16 h daylight regime with additional illumination with full spectrum phyto-lamps. The recombinant binary pEff-based expression vectors were transferred into *A. tumefaciens* strain GV3101 using electroporation. Recombinant *A. tumefaciens* strain was grown overnight with shaking at a temperature of 28 °C. The agrobacterial cells were collected by centrifugation for 5 min at 4000× *g*, and the pellet was resuspended in 10 mM MES (pH 5.5) and 10 mM MgSO_4_ to an OD600 of 0.2 and incubated for 3 h at room temperature. The suspension of agrobacteria was infiltrated into plant leaves using a syringe without a needle.

### 4.3. Protein Isolation and Analysis

The infiltrated leaves (8–10 mg) were homogenized in 50 µL of the PBS buffer (50 mM sodium phosphate pH 8.0, 300 mM NaCl). The resulting suspension was mixed with a half volume of the 3x sample loading buffer (40% glycerol, 4% SDS, 50 mM Tris pH 6.8, 1% bromophenol blue, 5% beta-mercaptoethanol), resulting in a total protein fraction for SDS-PAGE. Then, 10 µL of the obtained mixture (corresponding to about 1 mg of leaf tissue) was analyzed by SDS-PAGE. After electrophoresis, the gel was stained with One-Step Blue Protein Gel Stain (BIOTIUM, San Francisco, CA, USA).

The plant-produced HEV/GFP protein was purified on Ni-NTA resin (QIAGEN, Hilden, Germany) under native conditions. Four days after infiltration, the *N. benthamiana* leaves were homogenized in a PBS buffer (50 mM sodium phosphate, pH 8.0, 300 mM NaCl). The suspension was filtered through a paper filter and subjected to centrifugation at 14,000× *g* for 10 min; the supernatant was used as a soluble protein extract. The extract was mixed with Ni–NTA resin (QIAGEN, Hilden, Germany) equilibrated with PBS and incubated for 60 min. The resin was then washed with PBS containing 10 mM imidazole (twice) and with 20 mM imidazole (twice). The elution was carried out in 500 mM imidazole. After elution, the protein was dialyzed against PBS (1:100, 4 buffer changes) using 3.5 K MWCO Slide-A-Lyzer Mini (Thermo Fisher Scientific, Waltham, MA, USA).

For Western blotting, the proteins separated in the SDS-PAGE gel were transferred onto a Hybond-P membrane (GE Healthcare, Chicago, IL, USA) by semi-dry transfer using the Trans-Blot Turbo Transfer System (Bio-Rad Laboratories, Hercules, CA, USA). The membrane was blocked with a 5% (*w*/*v*) solution of dry milk in TBS-T (20 mM Tris pH 8.0, 150 mM NaCI, 0.1% Tween 20) buffer for 1 h at room temperature and subsequently incubated with primary antibodies for 1 h at room temperature. As primary antibodies, we used mouse monoclonal antibodies against the hexahistidine tag (1 mg/mL, used at a dilution of 1:1000) (Abcam, Cambridge, UK) with the rabbit anti-mouse antibodies conjugated with peroxidase (1 mg/mL, used at a dilution of 1:10,000) (Promega, Madison, WI, USA) and rabbit antibodies against GFP (2 mg/mL, used at a dilution of 1:10,000) with anti-rabbit antibodies conjugated with peroxidase) (1 mg/mL, used at a dilution of 1:10,000) (Promega, Madison, WI, USA). Then, the membrane was washed three times with TBS-T buffer (15 min at room temperature) and incubated with secondary antibodies conjugated with peroxidase (Promega, Madison, WI, USA) for 1 h at room temperature. Specific protein–antibody complexes were visualized using a Western Blot ECL Plus kit (GE Healthcare, Chicago, IL, USA) and chemiluminescence detector Fusion Solo X (Vilber, Eberhardzell, Germany).

Expression of HEV/GFP and GFP in leaves was visualized under UV illumination and was assayed fluorometrically with a Fluorat-02 fluorometer (Lumex, St. Petersburg, Russia), excitation at 390 nm, emission at 510 nm.

Electrophoresis under native conditions was performed in 1% agarose (Type II Medium EEO, Sigma-Aldrich, St. Louis, MO, USA) gel in TAE buffer (pH 7.5) supplemented with 5% glycerol and 1 μg/mL ethidium bromide.

### 4.4. Structural Analysis of Nanoparticles

Particle assembly was examined using a transmission electron microscope and atomic force microscope. Electron microscopy was performed on a JEM 1400 instrument (JEOL, Tokyo, Japan). The purified proteins were placed on carbon-formvar-coated copper grids (TED PELLA, Redding, CA, USA) and stained with 1% (*w*/*v*) uranyl acetate in methanol. Atomic force microscopy was performed using an Integra Prima microscope and Nova SPM v.4.0 software (NT-MDT, Moscow, Russia). The scanning was performed in the semi-contact mode using gold cantilever NSG01 (NT-MDT). Particle characteristics were determined using the NT-MDT Nova v. 1.06.26 software supplied with the instrument. The polydispersity index (PDI) was calculated as the square of the polydispersity (standard deviation/mean).

### 4.5. ELISA

ELISA plates were coated overnight at 4 °C with serial dilutions (starting from 10 μg/mL and finishing in 0.625 μg/mL) of purified HEV, HEV/GFP or HEV/4M2e proteins in sodium bicarbonate buffer pH 8.5. After a single wash with PBST (PBS with 0.05% Tween), plates were treated with a blocking buffer (0.2% (*w*/*v*) BSA in PBS) for 1 h at 37 °C. After a single wash with PBS, the plates were probed with monoclonal antibody specific for M2e or GFP for 30 min at 37 °C. After five washes with PBST, peroxidase-labelled secondary antibody solutions were added, anti-rabbit IgG (Promega, Madison, WI, USA) for GFP or anti-mouse IgG/IgA/IgM (P-RAM Iss, Imtek Ltd., Moscow, Russia) for M2e. After incubation with the conjugate for 30 min, wells were washed five times with PBST. Tetramethylbenzidine (Vector-BEST, Novosibirsk, Russia) was used as a substrate for horseradish peroxidase. The reaction was stopped by adding HCI (0.5 N) and the optical density was measured at 450 nm in a plate reader Multiskan FC (Thermo Fisher Scientific, Waltham, MA, USA).

## 5. Conclusions

- A recombinant fusion protein comprising truncated HEV capsid with GFP or four tandem copies of M2e peptide inserted at the Tyr485 position can be transiently expressed in plants at a high level using viral vector based on the PVX genome.
- The plant-produced fusion proteins in vivo formed VLPs displaying GFP and 4M2e.
- HEV coat protein can be used as a carrier for the presentation of large antigens (dozens to hundreds of a.a.).
- Plant-produced HEV particles displaying four copies of M2e peptide could be useful research tools for the development of recombinant vaccines against influenza.

## Figures and Tables

**Table 1:** VLP characteristics determined using transmission electron microscopy and atomic force microscopy.

Particles	HEV/GFP	HEV/4M2e
**Electron microscopy**		
Diameter (nm) ^1^	24 ± 8	21 ± 6
PDI	0.11	0.08
**Atomic force microscopy**		
Height (nm) ^1^	37 ± 14	19 ± 6
PDI ^2^	0.14	0.10
Height/diameter ratio ^1^	0.12 ± 0.02	0.21 ± 0.03

^1^ Mean ± standard deviation; ^2^ according to height values.

## Data Availability

Data are contained within the article and supplementary materials.

## Supplementary Materials

The following supporting information can be downloaded at: https://www.mdpi.com/article/10.3390/v16071093/s1, Figure S1: Solubility analysis of HEV/GFP protein expressed in *N. benthamiana* plants; Figure S2: Expression of HEV/GFP protein in *N. benthamiana* plants on different days post infiltration; Figure S3: Expression and solubility analysis of HEV/4M2e protein expressed in *N. benthamiana* plants; Figure S4: HEV/GFP and HEV/4M2e particle size distribution; Figure S5: HEV/GFP and HEV/4M2e particle height to diameter ratio.

## References

[1] Nooraei S., Bahrulolum H., Hoseini Z.S., Katalani C., Hajizade A., Easton A.J., Ahmadian G. (2021). Virus-like Particles: Preparation, Immunogenicity and Their Roles as Nanovaccines and Drug Nanocarriers. J. Nanobiotechnol..

[2] Zhang L., Xu W., Ma X., Sun X.J., Fan J.B., Wang Y. (2023). Virus-like Particles as Antiviral Vaccine: Mechanism, Design, and Application. Biotechnol. Bioprocess. Eng..

[3] Bhat T., Cao A., Yin J. (2022). Virus-like Particles: Measures and Biological Functions. Viruses.

[4] Pushko P., Pumpens P., Grens E. (2013). Development of Virus-like Particle Technology from Small Highly Symmetric to Large Complex Virus-like Particle Structures. Intervirology.

[5] Lee Y.T., Ko E.J., Lee Y., Kim K.H., Kim M.C., Lee Y.N., Kang S.M. (2018). Intranasal Vaccination with M2e5x Virus-like Particles Induces Humoral and Cellular Immune Responses Conferring Cross-Protection against Heterosubtypic Influenza Viruses. PLoS ONE.

[6] Wang C., Zheng X., Gai W., Wong G., Wang H., Jin H., Feng N., Zhao Y., Zhang W., Li N. (2017). Novel Chimeric Virus-like Particles Vaccine Displaying MERS-CoV Receptor-Binding Domain Induce Specific Humoral and Cellular Immune Response in Mice. Antivir. Res..

[7] Marsian J., Lomonossoff G.P. (2016). Molecular Pharming-VLPs Made in Plants. Curr. Opin. Biotechnol..

[8] Bachmann M.F., Jennings G.T. (2010). Vaccine Delivery: A Matter of Size, Geometry, Kinetics and Molecular Patterns. Nat. Rev. Immunol..

[9] Hemmati F., Hemmati-Dinarvand M., Karimzade M., Rutkowska D., Eskandari M.H., Khanizadeh S., Afsharifar A. (2022). Plant-Derived VLP: A Worthy Platform to Produce Vaccine against SARS-CoV-2. Biotechnol. Lett..

[10] Hills R.A., Howarth M. (2022). Virus-like Particles against Infectious Disease and Cancer: Guidance for the Nano-Architect. Curr. Opin. Biotechnol..

[11] Roldão A., Mellado M.C.M., Castilho L.R., Carrondo M.J.T., Alves P.M. (2010). Virus-like Particles in Vaccine Development. Expert. Rev. Vaccines.

[12] Tan M., Zhong W., Song D., Thornton S., Jiang X.E. (2004). Coli-Expressed Recombinant Norovirus Capsid Proteins Maintain Authentic Antigenicity and Receptor Binding Capability. J. Med. Virol..

[13] Aires K.A., Cianciarullo A.M., Carneiro S.M., Villa L.L., Boccardo E., Pérez-Martinez G., Perez-Arellano I., Oliveira M.L.S., Ho P.L. (2006). Production of Human Papillomavirus Type 16 L1 Virus-Like Particles by Recombinant Lactobacillus Casei Cells. Appl. Environ. Microbiol..

[14] Srivastava V., Nand K.N., Ahmad A., Kumar R. (2023). Yeast-Based Virus-like Particles as an Emerging Platform for Vaccine Development and Delivery. Vaccines.

[15] Gopal R., Schneemann A. (2018). Production and Application of Insect Virus-Based VLPs. Methods Mol. Biol..

[16] Rybicki E.P. (2020). Plant Molecular Farming of Virus-like Nanoparticles as Vaccines and Reagents. Wiley Interdiscip. Rev. Nanomed. Nanobiotechnol.

[17] Thuenemann E.C., Lenzi P., Love A.J., Taliansky M., Bécares M., Zuñiga S., Enjuanes L., Zahmanova G.G., Minkov I.N., Matic S. (2013). The use of transient expression systems for the rapid production of virus-like particles in plants. Curr. Pharm. Des..

[18] Mohsen M.O., Gomes A.C., Vogel M., Bachmann M.F. (2018). Interaction of Viral Capsid-Derived Virus-like Particles (VLPs) with the Innate Immune System. Vaccines.

[19] Shiri F., Petersen K.E., Romanov V., Zou Q., Gale B.K. (2020). Characterization and Differential Retention of Q Beta Bacteriophage Virus-like Particles Using Cyclical Electrical Field–Flow Fractionation and Asymmetrical Flow Field–Flow Fractionation. Anal. Bioanal. Chem..

[20] Zahmanova G., Takova K., Valkova R., Toneva V., Minkov I., Andonov A., Lukov G.L. (2022). Plant-Derived Recombinant Vaccines against Zoonotic Viruses. Life.

[21] Castells-Graells R., Lomonossoff G.P. (2021). Plant-Based Production Can Result in Covalent Cross-Linking of Proteins. Plant Biotechnol. J..

[22] Tusé D., Tu T., McDonald K.A. (2014). Manufacturing Economics of Plant-Made Biologics: Case Studies in Therapeutic and Industrial Enzymes. Biomed. Res. Int..

[23] Martínez C.A., Giulietti A.M., Rodríguez Talou J. (2012). Research Advances in Plant-Made Flavivirus Antigens. Biotechnol. Adv..

[24] Zahmanova G.G., Mazalovska M., Takova K.H., Toneva V.T., Minkov I.N., Mardanova E.S., Ravin N.V., Lomonossoff G.P. (2020). Rapid High-Yield Transient Expression of Swine Hepatitis E ORF2 Capsid Proteins in Nicotiana Benthamiana Plants and Production of Chimeric Hepatitis E Virus-Like Particles Bearing the M2e Influenza Epitope. Plants.

[25] Su H., van Eerde A., Rimstad E., Bock R., Branza-Nichita N., Yakovlev I.A., Clarke J.L. (2023). Plant-Made Vaccines against Viral Diseases in Humans and Farm Animals. Front. Plant Sci..

[26] Takova K., Koynarski T., Minkov G., Toneva V., Mardanova E., Ravin N., Lukov G.L., Zahmanova G. (2021). Development and Optimization of an Enzyme Immunoassay to Detect Serum Antibodies against the Hepatitis E Virus in Pigs, Using Plant-Derived ORF2 Recombinant Protein. Vaccines.

[27] Xing L., Kato K., Li T., Takeda N., Miyamura T., Hammar L., Cheng R.H. (1999). Recombinant hepatitis E capsid protein self-assembles into a dual-domain T = 1 particle presenting native virus epitopes. Virology.

[28] Li T.C., Yamakawa Y., Suzuki K., Tatsumi M., Razak M.A., Uchida T., Takeda N., Miyamura T. (1997). Expression and self-assembly of empty virus-like particles of hepatitis E virus. J. Virol..

[29] Avdjieva I., Terziyski I., Zahmanova G., Simeonova V., Kulev O., Krustev E., Krachunov M., Nisheva M., Vassilev D. (2019). Homology Based Computational Modelling of Hepatitis-E Viral Fusion Capsid Protein. Comptes Rendus L’Academie Bulg. Des Sci..

[30] Jariyapong P., Xing L., van Houten N.E., Li T.C., Weerachatyanukul W., Hsieh B., Moscoso C.G., Chen C.C., Niikura M., Cheng H.H.R. (2013). Chimeric Hepatitis E Virus-like Particle as a Carrier for Oral-Delivery. Vaccine.

[31] Mardanova E.S., Kotlyarov R.Y., Stuchinskaya M.D., Nikolaeva L.I., Zahmanova G., Ravin N.V. (2022). High-Yield Production of Chimeric Hepatitis E Virus-Like Particles Bearing the M2e Influenza Epitope and Receptor Binding Domain of SARS-CoV-2 in Plants Using Viral Vectors. Int. J. Mol. Sci..

[32] Sainsbury F., Lomonossoff G.P. (2008). Extremely High-Level and Rapid Transient Protein Production in Plants without the Use of Viral Replication. Plant Physiol..

[33] Marillonnet S., Giritch A., Gils M., Kandzia R., Klimyuk V., Gleba Y. (2004). In Planta Engineering of Viral RNA Replicons: Efficient Assembly by Recombination of DNA Modules Delivered by Agrobacterium. Proc. Natl. Acad. Sci. USA.

[34] Lindbo J.A. (2007). TRBO: A High-Efficiency Tobacco Mosaic Virus RNA-Based Overexpression Vector. Plant Physiol..

[35] Yamamoto T., Hoshikawa K., Ezura K., Okazawa R., Fujita S., Takaoka M., Mason H.S., Ezura H., Miura K. (2018). Improvement of the Transient Expression System for Production of Recombinant Proteins in Plants. Sci. Rep..

[36] Mardanova E.S., Blokhina E.A., Tsybalova L.M., Peyret H., Lomonossoff G.P., Ravin N.V. (2017). Efficient Transient Expression of Recombinant Proteins in Plants by the Novel pEff Vector Based on the Genome of Potato Virus X. Front. Plant Sci..

[37] Feng J.Q., Zhang M., Mozdzanowska K., Zharikova D., Hoff H., Wunner W., Couch R.B., Gerhard W. (2006). Influenza A Virus Infection Engenders a Poor Antibody Response against the Ectodomain of Matrix Protein 2. Virol. J..

[38] Kolpe A., Schepens B., Fiers W., Saelens X. (2017). M2-Based Influenza Vaccines: Recent Advances and Clinical Potential. Expert. Rev. Vaccines.

[39] Lim C.M.L., Komarasamy T.V., Adnan N.A.A.B., Radhakrishnan A.K., Balasubramaniam V.R.M.T. (2024). Recent Advances, Approaches and Challenges in the Development of Universal Influenza Vaccines. Influenza Other Respir. Viruses.

[40] Mardanova E.S., Ravin N.V. (2018). Plant-Produced Recombinant Influenza A Vaccines Based on the M2e Peptide. Curr. Pharm. Des..

[41] Neirynck S., Deroo T., Saelens X., Vanlandschoot P., Jou W.M., Fiers W. (1999). A Universal Influenza A Vaccine Based on the Extracellular Domain of the M2 Protein. Nat. Med..

[42] Feranmi F. (2022). Universal flu vaccine protects against influenza A and B. Lancet Microbe.

[43] Ravin N.V., Blokhina E.A., Kuprianov V.V., Stepanova L.A., Shaldjan A.A., Kovaleva A.A., Tsybalova L.M., Skryabin K.G. (2015). Development of a candidate influenza vaccine based on virus-like particles displaying influenza M2e peptide into the immunodominant loop region of hepatitis B core antigen: Insertion of multiple copies of M2e increases immunogenicity and protective efficiency. Vaccine.

[44] Ma W. (2020). Swine Influenza Virus: Current Status and Challenge. Virus Res..

[45] Vincent A.L., Perez D.R., Rajao D., Anderson T.K., Abente E.J., Walia R.R., Lewis N.S. (2017). Influenza A Virus Vaccines for Swine. Vet. Microbiol..

[46] Biasini M., Bienert S., Waterhouse A., Arnold K., Studer G., Schmidt T., Kiefer F., Cassarino T.G., Bertoni M., Bordoli L. (2014). SWISS-MODEL: Modelling Protein Tertiary and Quaternary Structure Using Evolutionary Information. Nucleic Acids Res..

[47] Poria R., Kala D., Nagraik R., Dhir Y., Dhir S., Singh B., Kaushik N.K., Noorani M.S., Kaushal A., Gupta S. (2024). Vaccine Development: Current Trends and Technologies. Life Sci..

[48] Eidenberger L., Kogelmann B., Steinkellner H. (2023). Plant-Based Biopharmaceutical Engineering. Nat. Rev. Bioeng..

[49] Rozov S.M., Permyakova N.V., Deineko E.V. (2018). Main Strategies of Plant Expression System Glycoengineering for Producing Humanized Recombinant Pharmaceutical Proteins. Biochemistry.

[50] Mardanova E.S., Takova K.H., Toneva V.T., Zahmanova G.G., Tsybalova L.M., Ravin N.V. (2020). A Plant-Based Transient Expression System for the Rapid Production of Highly Immunogenic Hepatitis E Virus-like Particles. Biotechnol. Lett..

[51] Pumpens P., Grens E. (2001). HBV Core Particles as a Carrier for B Cell/T Cell Epitopes. Intervirology.

[52] Kratz P.A., Böttcher B., Nassal M. (1999). Native Display of Complete Foreign Protein Domains on the Surface of Hepatitis B Virus Capsids. Proc. Natl. Acad. Sci. USA.

[53] Skamel C., Ploss M., Böttcher B., Stehle T., Wallich R., Simon M.M., Nassal M. (2006). Hepatitis B Virus Capsid-like Particles Can Display the Complete, Dimeric Outer Surface Protein C and Stimulate Production of Protective Antibody Responses against Borrelia burgdorferi Infection. J. Biol. Chem..

[54] Byrne M.J., Steele J.F.C., Hesketh E.L., Walden M., Thompson R.F., Lomonossoff G.P., Ranson N.A. (2019). Combining Transient Expression and Cryo-EM to Obtain High-Resolution Structures of Luteovirid Particles. Structure.

[55] Pleshakova T.O., Bukharina N.S., Archakov A.I., Ivanov Y.D. (2018). Atomic Force Microscopy for Protein Detection and Their Physicoсhemical Characterization. Int. J. Mol. Sci..

[56] Lampinen V., Heinimäki S., Laitinen O.H., Pesu M., Hankaniemi M.M., Blazevic V., Hytönen V.P. (2021). Modular Vaccine Platform Based on the Norovirus-like Particle. J. Nanobiotechnol..

[57] Wibowo N., Chuan Y.P., Lua L.H.L., Middelberg A.P.J. (2013). Modular Engineering of a Microbially-Produced Viral Capsomere Vaccine for Influenza. Chem. Eng. Sci..

[58] Zykova A.A., Blokhina E.A., Stepanova L.A., Shuklina M.A., Tsybalova L.M., Kuprianov V.V., Ravin N.V. (2022). Nanoparticles based on artificial self-assembling peptide and displaying M2e peptide and stalk HA epitopes of influenza A virus induce potent humoral and T-cell responses and protect against the viral infection. Nanomedicine.

[59] Tompkins S.M., Zhao Z.-S., Lo C.-Y., Misplon J.A., Liu T., Ye Z., Hogan R.J., Wu Z., Benton K.A., Tumpey T.M. (2007). Matrix Protein 2 Vaccination and Protection against Influenza Viruses, Including Subtype H5N1. Emerg. Infect. Dis..

[60] Kotlyarov R.Y., Kuprianov V.V., Migunov A.I., Stepanova L.A., Tsybalova L.M., Kiselev O.I., Ravin N.V., Skryabin K.G. (2010). Development of Recombinant Vaccine against A(H1N1) 2009 Influenza Based on Virus-like Nanoparticles Carrying the Extracellular Domain of M2 Protein. Acta Naturae.

[61] Katoh K., Standley D.M. (2013). MAFFT multiple sequence alignment software version 7: Improvements in performance and usability. Mol. Biol. Evol..

