# Supplementary material for: Plant-Produced Chimeric Hepatitis E Virus-like Particles as Carriers for Antigen Presentation
Source: Viruses. 2024 Jul 8;16(7):1093. doi: 10.3390/v16071093 (PMC11281382; doi:10.3390/v16071093)
Supplement: Supplementary file 1 [file viruses-16-01093-s001.zip › viruses-3039336-supplementary.pdf]

Supplementary Materials

Plant-Produced Chimeric Hepatitis E Virus-Like Particles as Carriers for Antigen Presentation

Eugenia S. Mardanova, Egor A. Vasyagin, Kira G. Kotova, Gergana G. Zahmanova, Nikolai V. Ravin

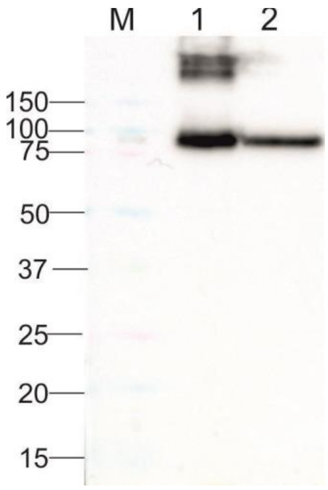

Figure S1. Solubility analysis of HEV/GFP protein expressed in *N. benthamiana* plants. Western blotting with antibodies against GFP. M, molecular weight marker (kD); 1, total proteins isolated from leaf infiltrated with pEff_HEV/GFP; 2, total soluble proteins isolated from leaf infiltrated with pEff_HEV/GFP.

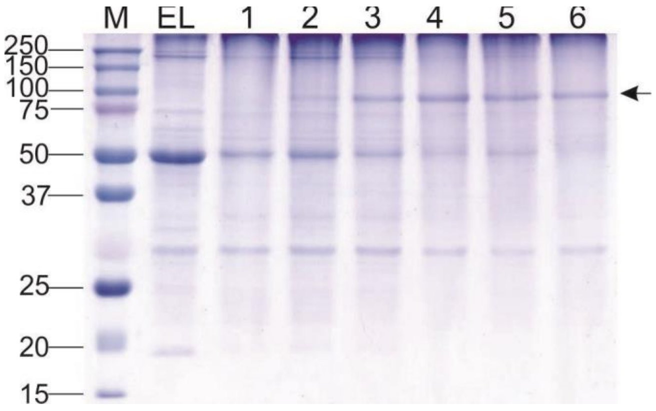

Figure S2. Expression of HEV/GFP protein in *N. benthamiana* plants on different days post infiltration. Coomassie brilliant blue-stained gel of proteins isolated from plants. M, molecular weight marker (kD); EL, total proteins isolated from non-infiltrated leaf; Lanes: 1–6, total proteins isolated from leaves infiltrated with pEff_HEV/GFP in 1–6 dpi, respectively. Position of HEV/GFP protein is shown by an arrow.

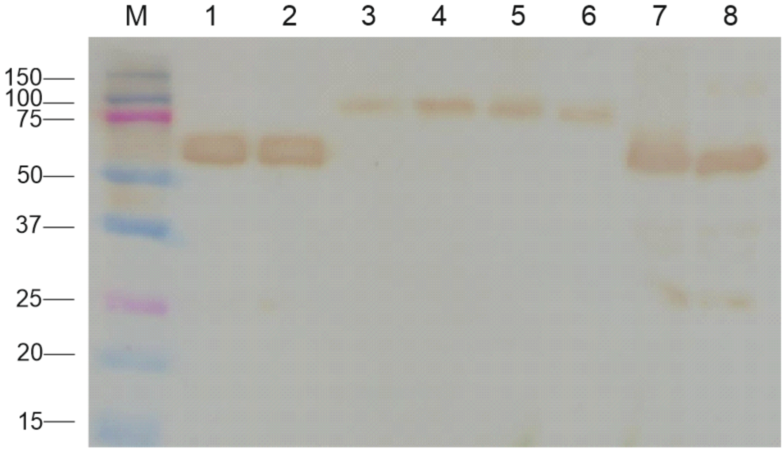

Figure S3. Expression and solubility analysis of HEV/4M2e protein expressed in *N. benthamiana* plants. Western blotting with antibodies against hexahistidine tag. M, molecular weight marker (kD); 1 and 2, soluble and total proteins isolated from leaf infiltrated with pAeff_HEV; 3 and 4, soluble fraction and total proteins isolated from leaf infiltrated with pAeff_HEV/4M2e; 5 and 6, soluble fraction and total proteins isolated from leaf infiltrated with pEff_HEV/4M2e; 7 and 8, soluble fraction and total proteins isolated from leaf infiltrated with pEff_HEV.

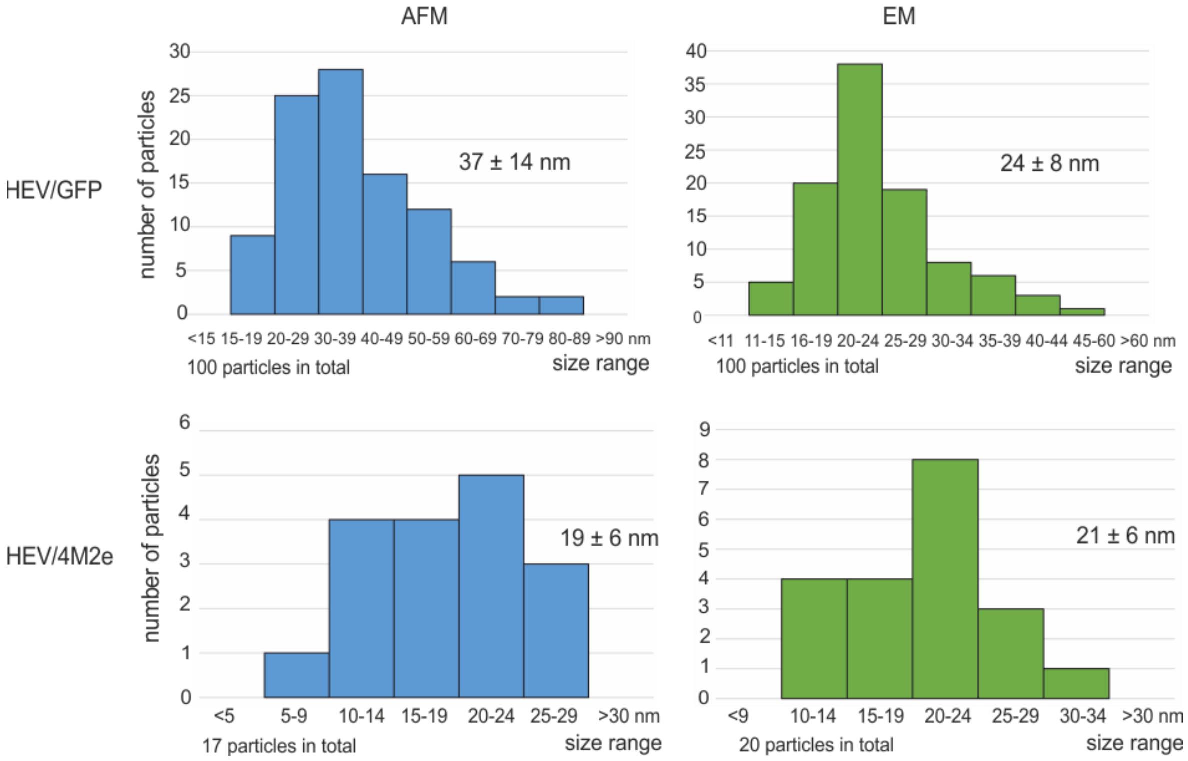

Figure S4. HEV/GFP and HEV/4M2e particle size distribution. The data were obtained using the atomic force microscopy (AFM) and transmission electron microscopy (EM) images. Mean ± standard deviation values are shown.

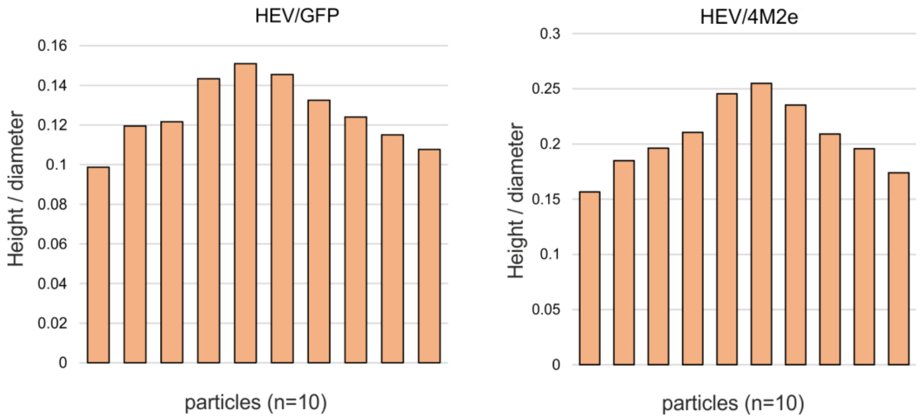

Figure S5. HEV/GFP and HEV/4M2e particle height to diameter ratio. The data are based on AFM images of 10 particles.
